# A systematic approach to managing complications after proximal tibial osteotomies of the knee

**DOI:** 10.1186/s40634-023-00708-7

**Published:** 2023-12-06

**Authors:** Jérôme Valcarenghi, Giulio Vittone, Caroline Mouton, Alexandre Coelho Leal, Maximiliano Ibañez, Alexander Hoffmann, Dietrich Pape, Matthieu Ollivier, Romain Seil

**Affiliations:** 1 Department of Orthopaedic Surgery, Centre Hospitalier de Luxembourg - Clinique d’Eich, Luxembourg, Luxembourg; 2 Department of Orthopaedic Surgery, Centre Hospitalier Universitaire d’Ambroise Paré, Mons, Belgium; 3 Department of Medical and Surgical Specialties, Radiological Sciences and Public Health, University of Brescia, Brescia, Italy; 4 Luxembourg Institute of Research in Orthopaedics, Sports Medicine and Science (LIROMS), Luxembourg, Luxembourg; 5 Department of Orthopedics and Traumatology, Hospital del Mar, Barcelona, Spain; 6 Institut Català de Traumatologia I Medicina de L’Esport (ICATME), Hospital Universitari Quiron-Dexeus, Carrer Sabino de Arana, 5-19, 08028 Barcelona, Spain; 7 Department of Orthopedics and Traumatology, Institute of Movement and Locomotion, St Marguerite Hospital, Marseille, France; 8 Department of Orthopedics and Traumatology, Institute for Locomotion, APHM, CNRS, ISM, Sainte- Marguerite Hospital, Aix Marseille University, Marseille, France; 9 Human Motion, Orthopaedics, Sports Medicine and Digital Methods (HOSD), Luxembourg Institute of Health, Luxembourg, Luxembourg

**Keywords:** Knee osteoarthritis, Osteotomy, Knee joint, Internal fixation, Joint-preserving surgery

## Abstract

Proximal tibial osteotomy (PTO) is an effective procedure for active and young adult patients with symptomatic unicompartmental osteoarthritis and malalignment. They were considered technically demanding and prone to various complications related to the surgical technique, biomechanical or biological origin. Among the most important are hinge fractures and delayed or non-healing, neurovascular complications, loss of correction, implant-related problems, patellofemoral complaints, biological complications and changes in limb length. Being aware of these problems can help minimizing their prevalence and improve the results of the procedure.

The aim of this narrative review is to discuss the potential complications that may occur during and after proximal tibial osteotomies, their origin and ways to prevent them.

## Introduction

One of the main reasons for the decrease in the popularity of knee osteotomies in the 1990s and 2000s was their high complication rate. Since these early days, limb realignment procedures using plate fixators have however dramatically evolved, mainly because of the development of new surgical techniques and internal fixators displaying a high primary stability and causing a low degree of correction loss [1]. This has led to a new expansion of the procedure and refinement of its indications while making conversions to arthroplasty less frequent and technically easier.

To guide and strengthen the widespread practice of osteotomy around the knee, the osteotomy committee of European Society of Sports Traumatology, Knee Surgery & Arthroscopy (ESSKA) recently published its European consensus [2]. According to this collaborative work, a complication following osteotomy surgery may be defined as a significant deviation from the normal course of events, either during surgery or during the post-operative period.

Complications may be classified as surgical-related, of biomechanical or of biological origin. Teitge and Van Heerwaarden have listed the possible complications after knee joint osteotomies (Table 1) [3], which displayed a high prevalence in older studies [4–6]. More recent publications showed a reduction of the number of clinically relevant complications to around or below the 5% -range (Table 2), which seems acceptable for this type of procedure.

**Table 1:** Known complications after knee osteotomies

Correction issue	Instability	Pseudarthrosis of the fibula
Overcorrection	Leg length modification	Patellofemoral pain syndrome
Fracture of the countercortical bone	Extension deficit	Infection
Pseudarthrosis/delayed bone healing	Genu recurvatum	Vascular complications (deep venous thrombosis,arterial injury)
Implant failure	Tibial plateau fracture	Extension deficit
Lesion of the peroneal nerve	Rotation malalignment	Compartmental syndrome

**Table 2:** Examples of reported complication rates before the era of plate fixation, compared with more recent work (without claim to completeness)

Authors	Publication’s year	Osteotomy’s type	Number of patients	Complications rate (%)
Surgical techniques (before plate fixation)				
Song et al	2010	PTO, laterally closing (clamps)	194	24
		PTO, medial opening (no plate fixation)		
Van den Bekerom et al	2008	/	40	62.5
Current surgical techniques (plate fixation)				
Flörkemeier et al	2013	PTO, medial opening	533	6
Palmer et al	2018	PTO, medial opening	528	3
Palmer et al	2018	DFO, medial closing	108	4
Niemeyer et al	2008	PTO, medial opening	46	4.3

*HTO* High Tibial Osteotomy, *DFO* Distal Femur Osteotomy

The aim of this narrative review is to discuss the potential complications that may occur during and after proximal tibial osteotomies (PTO), their origin and ways to prevent them. The rarer derotation, flexion, or extension osteotomies are not considered, nor are complex axis corrections with external fixators or major extra-articular corrections.

### Surgeons’ learning curve

One of the factors contributing to the complication rate is surgeon's experience, a sensitive variable that is difficult to quantify and is, therefore, rarely reported in the literature. In a study published in 2010 by Chae et al. [7], the authors achieved an unacceptably high complication rate of 60% the first year they started to perform PTO's in 2003. In the second year, it was reduced by almost half (32%), and in the following years, it continued to decrease (2006: 23.3%; 2007: 18.7%) until it fell below the acceptable 5% level in 2008. This highlights the importance of surgical experience and opens the debate about orthopedic surgeons’ adequate training time and the need for structured teaching curricula [8, 9].

Surgical experience should furthermore always be considered in the scientific interpretation of study outcomes. An example was this prospective randomized study published by Nerhus et al. [10]. Here, the results of lateral closing HTOs were compared with those of a medial opening technique. No difference was found regarding the clinical outcome or complication rate, which was however unacceptably high (33% in both groups). The authors did not discuss this high rate further but it can be assumed that surgeons' experience may have influenced both the results and the conclusions of the study [11]. These findings illustrate the need for orthopaedic surgeons to acquire the necessary theoretical and practical expertise before starting these procedures, be it either during or after orthopaedic residency.

Newly developed patient-specific cutting guides (PSCGs) could help surgeons in their learning phase to shorten the learning curve and limit complication rates. These are based on pre-operative CT-scan and allow for an accurate correction of both the frontal and the sagittal planes [12, 13]. They may provide a higher degree of accuracy when compared to conventional techniques as demonstrated both in vitro and in vivo, [13–16]. More importantly, using PSCGs is associated with a decrease in operating time (learning curve of 10 cases) and a reduction in the number of fluoroscopic images per procedure (learning curve of 9 cases) compared to conventional techniques [12, 13].

## Methods

Publications on complications after PTO available through PubMed database or the authors' personal library were reviewed. Complications are defined as undesirable outcomes that can be categorized as being either patient-related or procedure-related, and occurring either intra- or post-operatively. Long term scenarios such as total knee arthroplasty (TKA) after failure PTO or complex surgeries associated with associated injuries like ligament reconstructions or meniscus transplantations were excluded. Both prospective and retrospective studies were taken into consideration, without limits regarding follow-up length and number of patients studied. Studies on derotation, flexion, or extension osteotomies or complex alignment corrections with external fixators or major extra-articular corrections were excluded. Likewise, this narrative review does not consider complications due to significant planning mistakes, already described by Holschen and Lobenhoffer in 2016 [4].

## Intra-operative complications

### Hinge fractures/loss of primary stability

Osteotomies with plate fixation usually lead to bone healing after six weeks. Delayed healing can be related to the presence of a hinge fracture. Lateral hinge fractures (LHF) are one of the most common complications of medial opening wedge proximal tibial osteotomy (MOWPTO), and are the leading cause of instability, displacement, non-union, and varus recurrence after this procedure [17, 18]. They can hardly be avoided with the current surgical techniques, even if the procedure is correctly performed with no technical mistakes.

The incidence of LHF based on radiographic reports varies between 3 and 30%. Pape et al. has shown that, for an opening tibial osteotomy gap of 8°, such a fracture occurs in up to 75% of patients [19]. Whether and when these hinge fractures can be considered surgical complications is controversial. In many cases, LHF can be diagnosed intraoperatively with the help of an image intensifier. They may also occur postoperatively, with incidence rates up to 18% [20].

#### Diagnosis and Classification

LHF may be underestimated with intraoperative fluoroscopy or immediate postoperative radiographs [20, 21]. They are best diagnosed and classified with CT scans [20]. Recent computer tomography (CT)-based studies have shown that their prevalence ranges from 13.8 to 27.5% (Table 3) [2, 5, 15, 18].

**Table 3:** Analysis of counter-cortical fractures in medial opening high tibial osteotomy (PTO) and fixation with plate fixation

Authors	Number of osteotomies	Imaging	Number of countercortical fractures (n, %)	Fracture type according to Takeuchi (n, %)		
				**I**	**II**	**III**
Chaouche et al	100	CT after 4 weeks	25 (25)	18 (18)	3 (3)	3 (3)
Lee et al	51	CT after 2 days	14 (27.5)	11 (21.5)	2 (3.9)	1 (1.9)
Kim et al	164	CT preoperatively, 6 weeks, 3, 6, 12 months postoperatively	37 (22.6)	16 (9.8)	21 (12.8)	0
Lee u. Lee	94	X-ray, postoperative CT	23 (24)	14 (14.9)	3 (18.1)	6 (6.4)

**Fig. 1:**
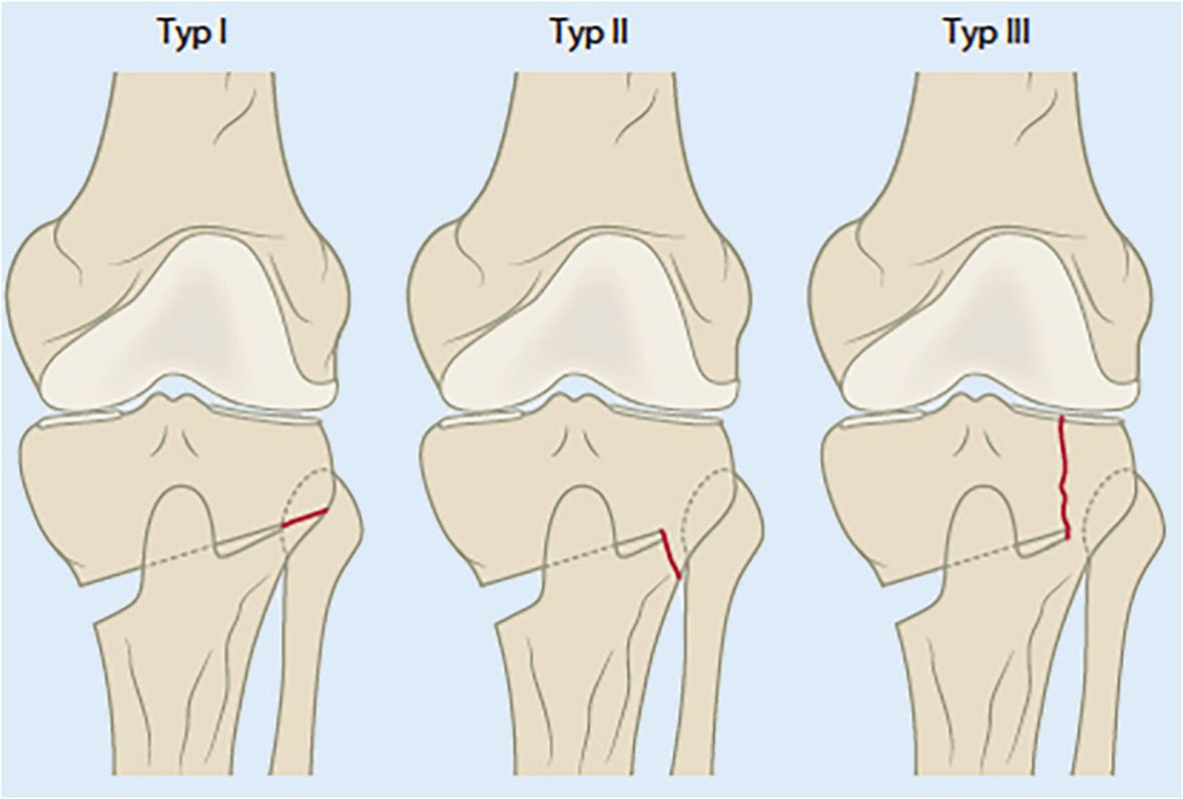
Hinge fractures in medial opening wedge proximal tibial osteotomy (PTO). Classification according to Takeuchi et al. [22]: Type I: at the level of the tibiofibular joint, Type II: below the tibiofibular joint, Type III: lateral tibial plateau fracture. (From [11]with kind permission)

Takeuchi et al. distinguished between 3 types of hinge fractures after medial opening PTO (Fig. 1) [22]. Type I fractures affect only the lateral cortex of the proximal tibial metaphysis (Fig. 1) and involved about two-thirds of all hinge fractures. They usually have a benign course and heal without consequences [17, 18]. If the fracture of the opposite cortex is oriented distally (type II), it can lead to high-grade instabilities of the osteotomy leading to painful pseudarthrosis or delayed bone healing (Fig. 1). They can easily be overlooked intraoperatively and usually heal without consequences if treated correctly [5, 17]. If necessary, the non-weight-bearing phase may be extended beyond six weeks. In very rare cases, revision surgery with debridement and cancellous bone grafting may become necessary. Schröter et al. reported reduced osteotomy healing in the presence of a Takeuchi type II fracture. After six weeks, healing was noted in 57 ± 18% of patients with type II fracture compared with 73 ± 18% in patients without type II fracture [23]. The loss of primary fixation stability occurring in Takeuchi type II fracture often results in pain at weight-bearing after the 6-week unloading period. However, this can also be seen as a part of the typical clinical course in 38% of patients after medial opening PTO using a TomoFix plate fixation system (Depuy-Synthes, Oberdorf, Switzerland) [24].

**Fig. 2:**
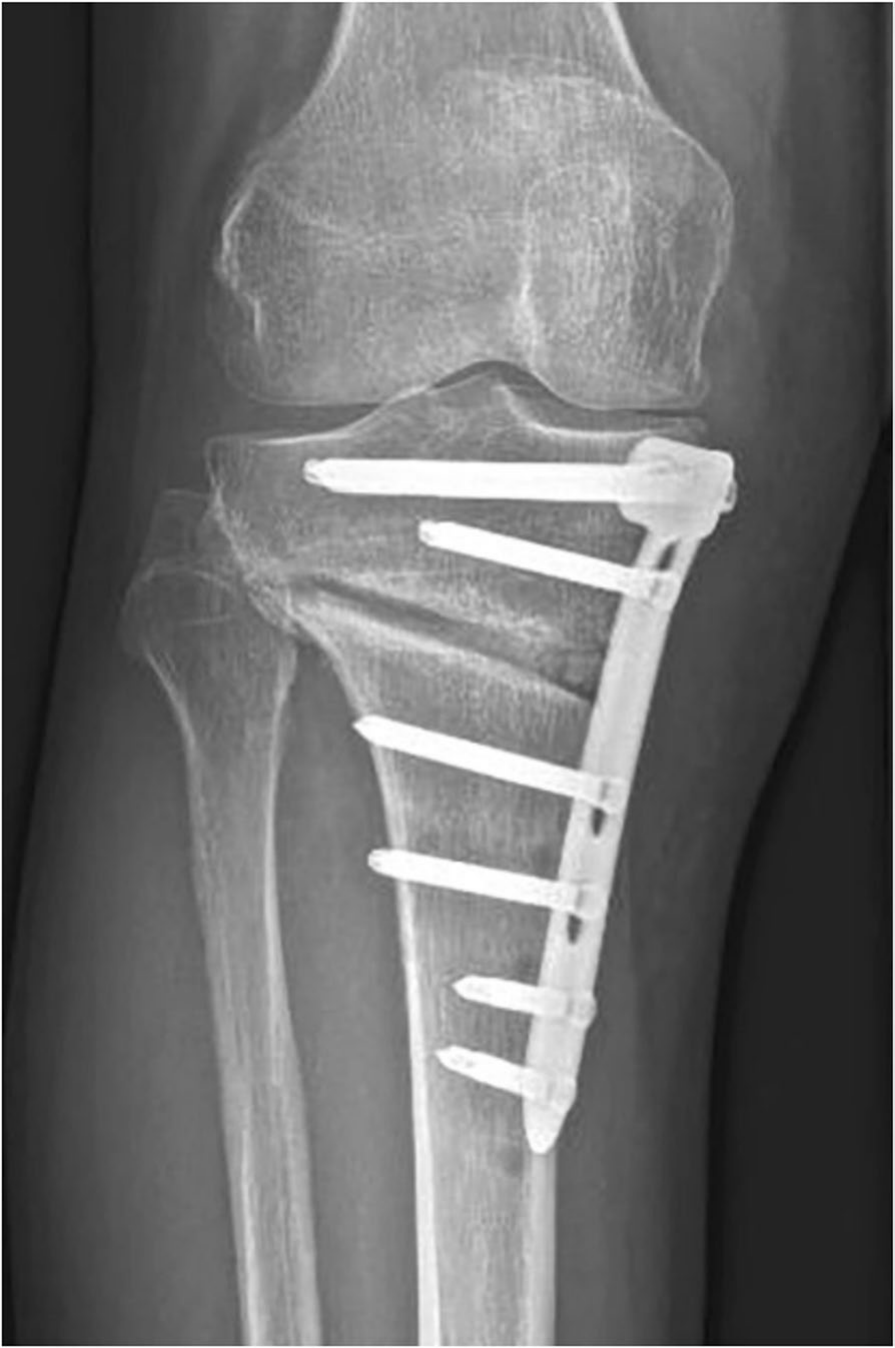
Lateral widening of the osteotomy gap at 6-week follow-up. This imaging finding should prompt a CT scan (e.g., to rule out a grade II Takeuchi unstable fracture)

**Fig. 3:**
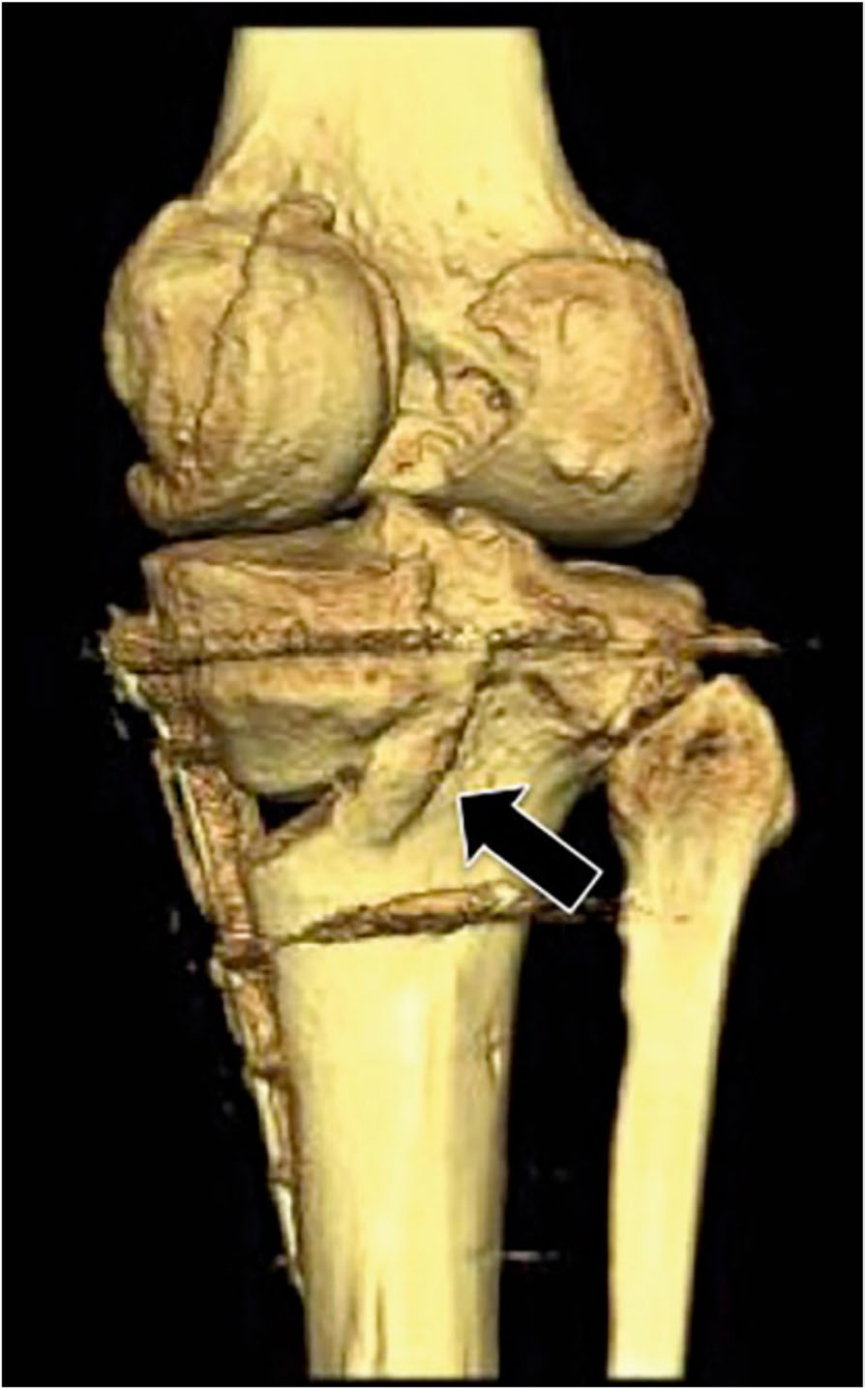
The presence of posteromedial callus formation on CT scans at six or more weeks postoperatively represents an excellent prognostic factor (arrow)

Further diagnostic clarification is required in front of pronounced and persisting complaints after full weight bearing. Compared to the postoperative radiographic control, a CT scan may be performed to exclude loss of correction or even a laterally extending widening of the osteotomy gap (Fig. 2). Modern CT techniques allow metal artifacts to be kept to a minimum and a reliable judgment to be made. Posteromedial bone callus formation on CT scans after six or more weeks is a good prognostic factor (Fig. 3).

Particular caution is advised in patients with a type II fracture and another risk factor for delayed bone healing, such as smoking [25].

**Fig. 4:**
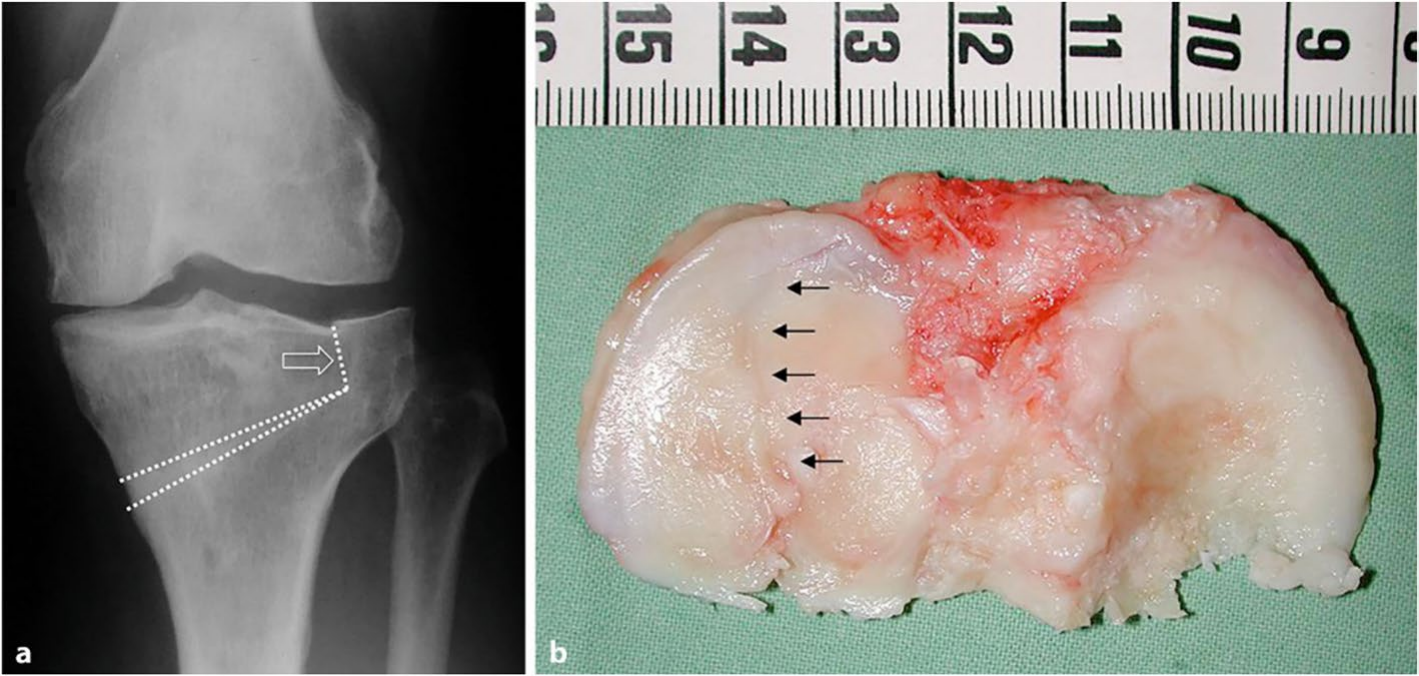
**a.** Postoperative radiograph of a type III fracture protruding proximally into the lateral joint space.Takeuchi type III fracture (arrow) after medial opening wedge proximal tibial osteotomy (PTO) in a 44-year-old female patient. **b.** Tibial plateau cut after conversion to total knee arthroplasty five years after PTO. The arrows represent the still visible fracture line through the lateral tibial plateau

Takeuchi type III fractures may lead to rapid progression of osteoarthritis in the affected lateral tibiofemoral compartment and may require early conversion to total knee arthroplasty. These fractures are ascending proximally into the lateral joint space (Figs. 1 and 4). If they occur intraoperatively, additional stabilization by internal fixation, e.g., internal fixation screws, may be considered. If the diagnosis occurs postoperatively, the non-weight-bearing phase should be prolonged and the patient informed about the possible need for revision surgery. Given the current evidence, systematic revision surgery cannot be recommended at the time of diagnosis.

#### Prevention

The first step in reducing the prevalence of hinge fractures in MOWPTO is to correctly determine the height of the osteotomy in the frontal plane and to perform it within the considered “safety zone” [7]. The rule of thumb is to target the proximal third of the fibular head obliquely, proximally and laterally with the first Kirschner wire used as a template, starting from the concavity of the medial tibial metaphysis. It must be noted that the height of the fibular head is variable and does not always end at about 1 cm below the tibial joint line [26].

The osteotomy should be opened or closed with great care, slowly and progressively. The elasticity of the bone must be assessed by the surgeon on a case-by-case basis. If there is a lack of flexibility when opening the osteotomy, the depth of the bone cut should be checked to evaluate whether it is sufficient and avoid placing abrupt and excessive stress on the opposite cortex during the correction. In the case of MOWPTO, excessive ligament tension through the superficial medial collateral ligament, which bridges the osteotomy gap, must be excluded.

#### Management

Intraoperatively, the integrity of the opposite cortex should be systematically checked through an image intensifier. If the osteotomy opens or closes too easily, an unstable fracture should be suspected. In such cases, it is advantageous to use a fixation plate offering the possibility to compress the lateral hinge. In recent years, several surgical variations have been described to strengthen the hinge of the osteotomy and avoid fractures of the opposing cortex. A hole maybe drilled in the anteroposterior direction to relieve bending forces in the hinge area [27]. Recent biomechanical studies have however shown that this approach is only of minimal benefit at small correction angles [27]. A alternative is to temporarily stabilize the lateral tibial hinge with a Kirschner wire [20, 28, 29].

Various techniques to protect the hinge point have been described in the literature. Jacobi et al. [30] described the use of 5 wires in defined directions. In a retrospective cohort of 206 patients, Gulagaci et al. [29] showed that the addition of a K-wire at the level of the hinge could significantly reduce the stresses going through this point and reduce the incidence of a hinge fracture from 43.3%, to 16.7% [29]. The authors also demonstrated that using a protective K-wire shortens time to bone union, to return to work and/or to sport. A recent paper from Koh et al. [31] furthermore demonstrated that the use of a protective wire was associated with a low incidence of LHF even in larger PTO corrections (≥ 10 mm).

Likewise, adding a protective screw improves the axial and torsional stability of the hinge. In a 3D model simulation, Jacquet et al. [20] showed that positioning a 4 mm screw intersecting the cutting plane at the theoretical lateral hinge location associated with a locking plate reduces lateral hinge stress in both compression and torsion, which should reduce the incidence of fractures in this area during the consolidation phase. These results must however be confirmed by standard mechanical tests in vitro.

### Iatrogenic deformities in the sagittal plane

In a PTO, an iatrogenic extension deficit can occur if the angle between the horizontal and ascending osteotomy is too shallow in the case of biplanar osteotomy or if intraoperative care is not taken to achieve the full extension position at the moment of internal fixation [2]. In the case of medial opening PTO using the biplanar technique, care should also be taken to keep the sagittal inclination of the tibial plateau ("tibial slope") neutral. To maintain control over the tibial slope, the osteotomy spreader should be placed at the dorsomedial edge of the tibia at the moment of osteotomy opening. Ventral placement of the spreader leads to an increase of the tibial slope [2].

### Neurovascular complications

Wootton et al. reported 20% peroneal nerve damage after lateral closing PTO. The authors divided the fibula into four zones [32]. Zone 1 corresponds to the fibular head, zone 2 starts at the lower end of the fibular head and extends 7 cm distally, zone 3 involves the next 8–15 cm and zone 4 involves the distal portion of the fibula. Among these, the highest risk of intraoperative nerve damage has been reported in zones 2 and 3. Aydoglu et al. divided the fibula into three identical thirds and found the lowest risk at the transition from the middle to the distal third [33]. In the case of fibular osteotomy, overly aggressive electrocoagulation should be avoided.

In a recent systematic review, Miltenberg et al. reported that the most common intraoperative complication was a lateral hinge fracture (incidence, 9.1%; range, 0%-30.4%) in medially based PTO's and peroneal nerve injury in laterally based PTO's (incidence, 3.2%; range, 0%-8.7%). The overall incidence of neurovascular injury after medially or laterally based PTO's was 1.1% (range, 0%-18.9%) [34].

Gerich et al. published a review paper on rare vascular injuries after PTO [35]. In general, these were isolated cases with popliteal artery injuries or pseudoaneurysms. Interestingly, the moment of injury diagnosis was described from intraoperative to 3 years postoperatively. In case of persistent postoperative posterior pain, a proper imaging protocol of the popliteal artery should be considered to exclude a pseudoaneurysm.

In order to avoid additional vascular injuries, one particular surgical reference to take into account is the course of the anterior tibial artery, which usually runs dorsally from and at the level of the popliteus muscle before it breaks through the interosseous membrane. In some patients, this branch runs more proximally than usual, which may cause the artery to run ventral to the popliteus muscle. In these group, there is risk of lesion when sawing through the dorsal tibial cortex. If this occurs and is detected intraoperatively, the osteotomy incision should be opened and immediate hemostasis can be performed.

### Implant related

Plate positioning should be as accurate as possible. Excessive proximal placement may cause intra-articular interference with the plate or screws, while a too distal placement causes interposition of the proximal screws into the osteotomy space and therefore may cause delayed bone healing.

**Fig. 5:**
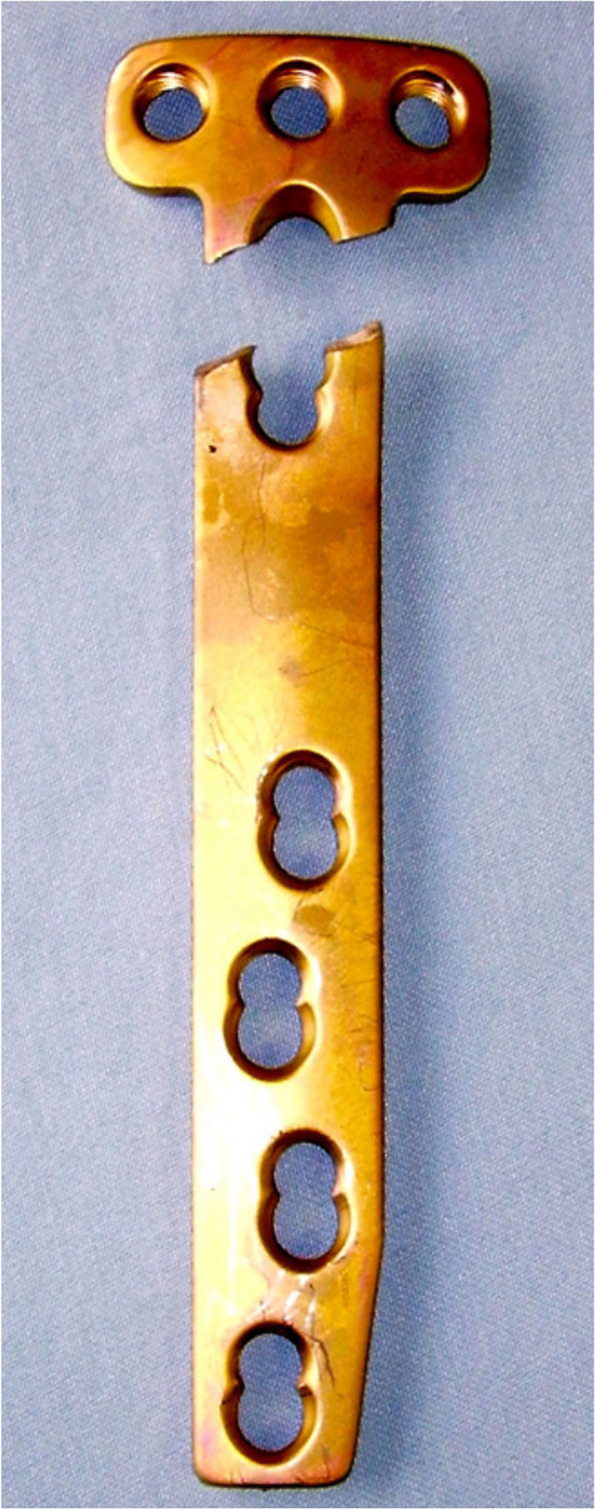
Fracture of a plate fixator (Tomo-Fix, Depuy-Synthes) due to lack of occupation of the "D-hole". This has led to too high a load concentration on the plate in this area and ultimately produced the plate fracture

**Fig. 6:**
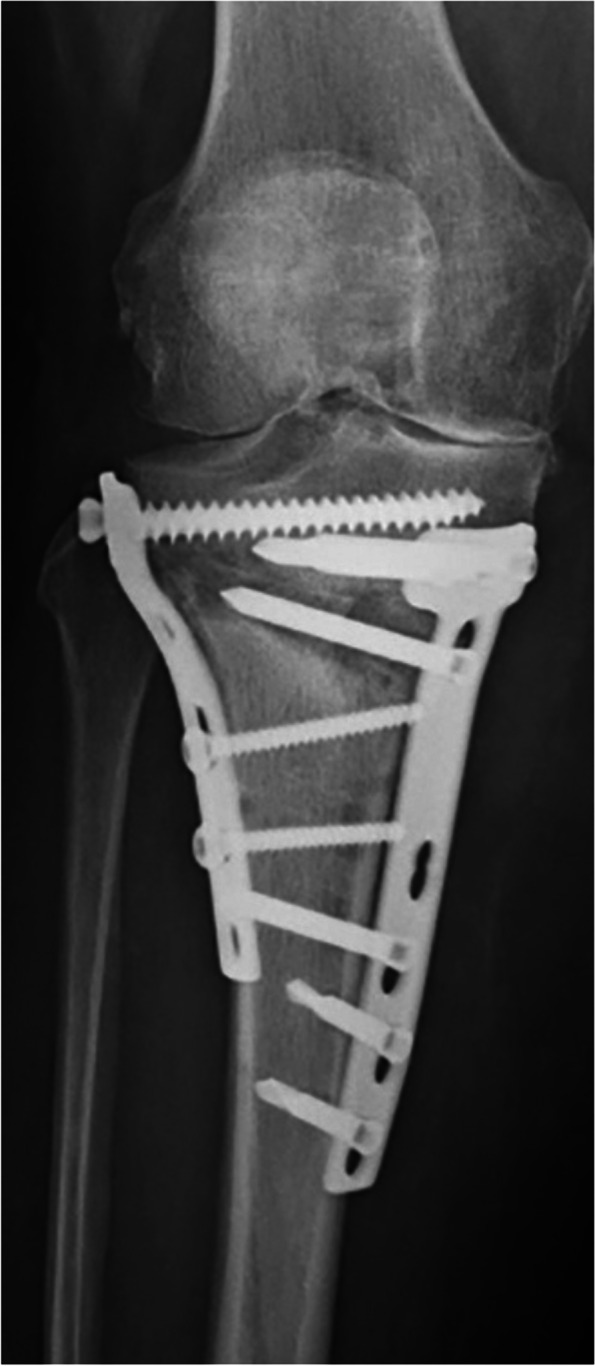
Revision double plate osteosynthesis and autologous bone grafting in patient after plate rupture

Implant failures are infrequent with modern plates. When they occur, they are usually the result of poor technical execution of the procedure. An example is plate insufficiency when the "D-hole" of the T-shaped Tomofix plate is not used. This leads to high load concentration on the plate in this area and, at the end, can cause fatigue and breakage of the material [36] (Fig. 5). Screw breakage can also be the cause of symptomatic postoperative evolutions [7]. In case of a clinically or radiographically detected loss of correction, implant failure must be ruled out. Revision surgery with internal revision fixation and bone grafting shall be considered in these cases. Double plating can be recommended, depending on the extent of the bony dislocation (Fig. 6).

## Post-operative complications

### Overcorrection

**Fig. 7:**
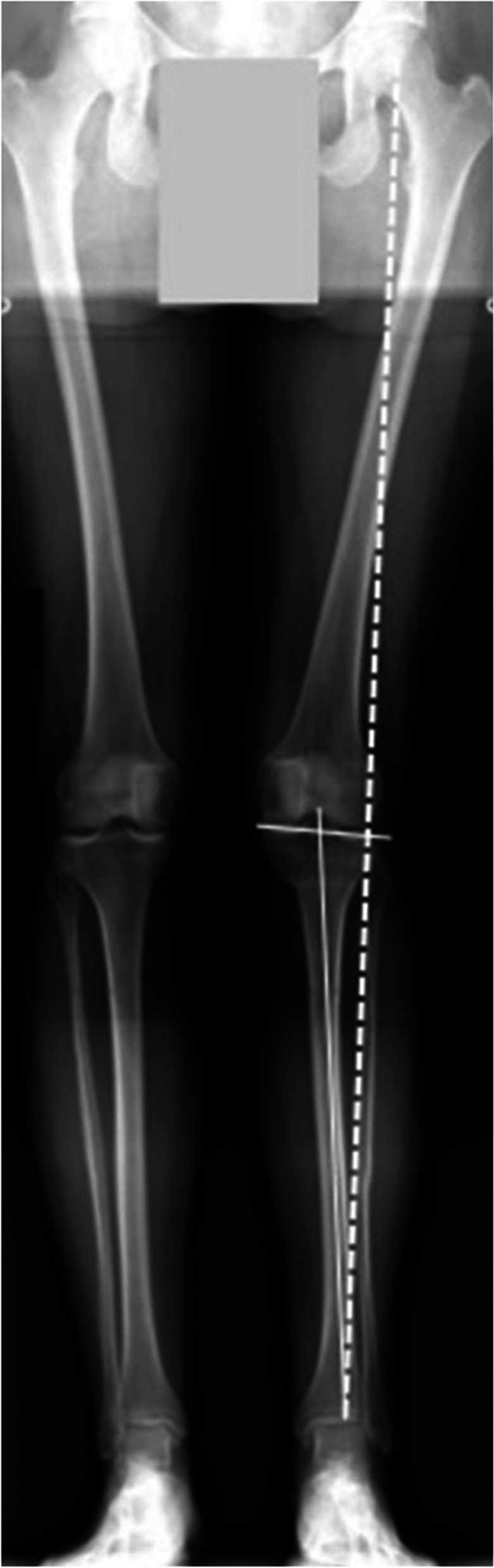
Postoperative long-leg radiograph of a 48-year-old female patient after left lateral closing wedge proximal tibial osteotomy (PTO) with overcorrection. The Mikulicz line passes far laterally through the lateral joint compartment (dashed line). There was also a laterally sloping joint line that exceeded the generally accepted 5° (solid lines)

A frequent scenario after PTO is an excessive correction angle in the coronal plane (Fig. 7).

In most patients, the proximal tibia displays a preoperative medial slope inclination of 3°, with a medial proximal tibial angle (MPTA) of 87° [2]. Overcorrection may lead to an excessive inclination of the joint line and an unphysiological loading of the lateral tibiofemoral compartment, generating pathological shear forces and lateral knee pain, despite a good overall correction of limb alignment.

The preoperative planning should not only involve the mechanical axis but also differentiate bony and intra-articular deformities due to osteoarthritis and soft tissue laxity [37]. By not considering the joint line convergence angle (JLCA), there is an increased risk of overcorrection.

The JLCA has become an important measure to analyze the long leg alignment views for corrective planning before osteotomy surgery. It is defined as the angle between the tangent to the most distal part of the medial and lateral femoral condyle and the subchondral plate of the tibial plateau on anteroposterior weightbearing long-leg radiographs [38].

This value represents the interaction of both, surrounding soft tissue and thickness of the remaining cartilage, and it can be influenced by the amount of ligament stretching or lateral soft tissue laxity during weight-bearing.

Clinical and experimental studies have shown that the JLCA has a normal range between 0° to 2°, which increases in magnitude depending on the severity and stage of osteoarthritis in the knee [1, 2, 10, 37, 39]. A positive value may be caused by an intra-articular varus deformity with a medial apex [40]. Park et al. showed that preoperative JLCA values greater than 4° or 1.5° of valgus stress angle were correlated with a greater risk of overcorrection [41].

When a correction of the MPTA of more than 95º was considered, higher JLCA and worse knee function could be found [42]. This PTO overcorrection can result in knee joint line obliquity (KJLO) increase, particularly when there is a large correction [43]. In a 3D finite element analysis of significant corrections for severe varus, Nakayama et al. [44] demonstrated that if the correction of a severe varus deformity was performed by a single level PTO, increased shear stresses were observed across the articular cartilage. Such increase was not observed if the correction was conducted both across the femur and the tibia. The authors conclude that an increased KJLO of more than 5 degrees may induce detrimental shear stresses across the articular cartilage.

To take into account the influence of soft tissue laxity to prevent overcorrection in cases with JLCA values > 2º, Micicoi et al. [37] proposed a simple equation “JLCA-2/2” to estimate the value to subtract from the planned correction.

A recent study by Behrendt et al. [39] provided the first evidence that this additional planning step improves the accuracy of varus correction osteotomies and yields reduced postoperative medial proximal tibial angle (MPTA) values.

In those cases where an overcorrection could not be prevented, either a new corrective osteotomy or an early conversion to a total knee arthroplasty may be performed to reduce the extent of the deformity.

### Patellofemoral complaints

The patellofemoral joint can be affected by a PTO. The courses of the patella can indeed shift in the sagittal and coronal planes and be influenced by rotational changes [34, 45]. A medial opening PTO decreases the height of the patella, while a lateral closing PTO may increase it [46, 47]. In most cases these changes are asymptomatic [46]. If patellofemoral complaints do nevertheless occur after adequate rehabilitation, a secondary patella infera should be ruled out. Iatrogenic patella infera can be avoided by cutting the tibial tuberosity distally using a biplanar technique [48]. In this case, the tuberosity should be reinforced with a screw to prevent avulsion due to excessive traction during quadriceps activation.

### Biological aspects

The biological aspects can be divided into preoperative risk factors and complications related to the surgical procedure itself.

#### Preoperative risk factors

Smoking has a negative effect on bone healing, which is why the indication for osteotomy should be critically reviewed in patients who experience difficulties to quit smoking several months before the procedure [49, 50]. At the very least, the patient must be aware of the possibility of delayed bone healing or pseudoarthrosis and accept a prolonged unloading phase or the risk of a second surgery [51].

Another relative contraindication is obesity. Although no clear threshold has been set yet for the body mass index (BMI) at which the procedure should be contraindicated, obese patients must be aware of the increased complication rates. These patients are most likely to experience a higher rate of infection or delayed bone healing.

#### Biological complications

Infections after osteotomies around the knee are rare (less than 1%) in centers with extensive experience. Infection rates can however vary between 0.8 and 17% [21, 24, 34, 52–56]. If the infection occurs after bone healing, the osteotomy plate should be removed, the wound cleaned, and germ-appropriate antibiotic therapy should be carried out. If the infection occurs before bone healing, at least one attempt should be made to eradicate the infection with the plate in place [57, 58]. If unsuccessful, a temporary fixation must be considered using an external fixator or plaster cast immobilization. Before making this decision, the authors recommend a systematic assessment of bone healing through CT scans to find any existing bone bridges, for example, in the area of the lateral hinge or the tibial tuberosity.

To avoid any thrombotic event, due to the prolonged non-weightbearing or partial weightbearing phase, thromboembolic prophylaxis must be performed for several weeks.

Soft tissue complications are more common if a cutaneous scar is already present. As with other knee procedures, this must be included in the preoperative considerations. In order to simplify a possible later conversion to a knee prosthesis, the authors recommend that the incision should not be made obliquely in either the medial or the lateral PTOs. Oblique scars may intersect the longitudinal incision required for a knee arthroplasty, leading to possible vascular disturbances of the skin and subcutaneous tissue after reoperation.

Finally, venous and lymphatic return from the lower leg may be altered for several months after realignment procedures and lead to prolonged swelling or, in the worst case, cellulitis or erysipelas. Adequate compression treatment and/or lymphatic drainage manipulation can significantly improve the symptoms.

### Hardware removal

Except for external fixation methods, maintenance of the PTO correction is traditionally completed with a metal plate. These plates have been shown to have drawbacks, especially hardware irritation by pressing on nearby structures, including the pes anserinus and hamstring tendons, the medial collateral ligament, and the overlying subcutaneous tissue and skin, necessitating subsequent plate removal [59].

A study by Niemeyer et al. [60] reported a 41% rate of irritation, with reported rates of hardware removal as high as 60% to 99% [60, 61]. In a more recent retrospective study, Sidhu R. et al. [62] reported that 52% of the patients required elective hardware removal due to soft tissue irritation. Darees et al. [63] report similar values of locking plate removal in 27 of the 48 patients (56.2%) after a mean of 30.71 months at ten years follow-up.

Additional disadvantages of a metal plate include loss of detail on MRI images and increased complexity of revision surgery, including the need to remove hardware for future joint arthroplasty. Recent polyether ether ketone (PEEK) implants have a reduced risk of hardware removal compared to metallic fixation. Hevesi et al. compared PTOs performed with metallic and PEEK implants between 2000 and 2015 in two institutions with a minimum follow-up of 2 years. They reported that both metal and PEEK implants were effective in obtaining and maintaining correction of coronal varus deformity, with an overall arthroplasty-free survival of 88% at 5 years [64].

### Loss of correction

Recurrent varus deformity is one of the common complications after PTO and can lead to the progression of medial joint arthritis and patient dissatisfaction [34, 65]. A 15.22% incidence of loss in angular correction was reported by Miller et al. related to a high BMI (average 32.5 [6]. A recent systematic review found an incidence of varus recurrency of 1.2% (range, 0%-34.3%). Similar results were published by Martin et al. with a loss of correction and hardware failure in 1% of the patients [5]. The gap created in the proximal tibia facilitates instability and varus recurrence due to lack of primary stability and predisposes this loss of correction [17]. Another key factor is the presence of hinge fractures and not due to plate or screw fixation failure [66].

Stability of the construct, including type of fixation with or without bone grafts or substitutes are the most important factors for maintaining the correction until the osteotomy healing. Goshima et al. suggest that plates can be safely removed without loss of correction when the posterior cortex bone union reaches the osteotomy gap center even if the total gap is incompletely filled [67].

Rates of failure of nonlocking plates have been reported between 3.6% [68] and 6.1% [17], but no such failures have been reported with the use of a locking plate [23]. Fixed-angle locking plates are thought to provide more stable fixation decreasing the complications of nonunion and failure of the osteotomy site, even without bone graft or substitutes [18].

In case of a lateral hinge fracture, if a non-locking plate has been used, additional stabilization through lateral plate or staple is required [69]. However, enough axial and torsional stability is found when a locking compression plate is used to allow partial weightbearing without the need for additional fixation [66].

### Limb length modifications

Limb length can be altered after PTO [70]. Increases after opening wedge PTO due to enlargement of the osteotomy site and decreases after closing wedge PTO due to bone loss at the osteotomy area have been reported, but the clinically significant discrepancy is still a matter of debate [71]. In a systematic review, the authors found that in most of the studies, the change in leg length was smaller after a closing-wedge PTO (CWPTO) than after an opening-wedge PTO (OWPTO). A higher number of patients reported discomfort and were aware of the leg length discrepancy after OWPTO compared to CWPTO [72].

Limb length discrepancies (LLD) may cause long term osteoarthritis, scoliosis, and gait abnormalities [73]. Hinarejos et al. reported that the clinically significant LLD lies at 10 mm [45], while other authors reported that differences of more than 20 mm can be compensated [74]. An attempt was made to generate a mathematical model allowing for patient-specific estimation of limb length change. The authors concluded that limb lengthening after OWPTO was statistically significant, but its clinical significance was minimal and further studies would be required to assess other influencing factors. However, the development of this reliable mathematical model may be useful in predicting the anatomical outcomes, improve patient expectations of anatomical results, and contribute to better patient satisfaction post-PTO [71].

## Conclusions

This narrative review provides a detailed overview of the possible complications that can take place at any stage of a PTO. Most of them can be prevented by proper patient selection, surgical planning, and surgical technique. Intra operative adverse outcomes such as hinge fractures, neurovascular and implant-related problems are usually due to incorrect planning, indication and technical execution. Post operative complications like overcorrections can be prevented by thorough surgical planning, taking into account not only the mechanical axis in the coronal plane, but also intra-articular deformities, as expressed by the JLCA and JLO. Special care should be taken when indicating an osteotomy in heavy smokers. In case of infection, aggressive treatment should always be carried out, assessing whether the hardware material should be removed or left in place. Loss of correction should be avoided, as progression to medial osteoarthritis and patient dissatisfaction is common, with incidences of up to 34%.

## Abbreviations

BMI: Body mass index
CT: Computer tomography
ESSKA: European Society of Sports Traumatology, Knee Surgery & Arthroscopy
PTO: Proximal tibial osteotomies
TKA: Total Knee Arthroplasty
MOWPTO: Medial opening wedge proximal tibial osteotomy
PEEK: Polyether ether ketone
MPTA: Medial proximal tibial angle
PSCGs: Patient-specific cutting guides
JLCA: Joint line convergence angle
KJLO: Knee joint line obliquity
LHF: Lateral hinge fractures
CWPTO: Closing-wedge PTO
OWPTO: Opening-wedge PTO
LLD: Limb length discrepancies
